# Intravenous Lipid Emulsion Therapy for Acute Synthetic Cannabinoid Intoxication: Clinical Experience in Four Cases

**DOI:** 10.1155/2015/180921

**Published:** 2015-05-11

**Authors:** Gökhan Aksel, Özlem Güneysel, Tanju Taşyürek, Ergül Kozan, Şebnem Eren Çevik

**Affiliations:** ^1^Umraniye Training and Research Hospital, Emergency Medicine Clinic, Istanbul, Turkey; ^2^Dr. Lutfi Kirdar Kartal Education and Research Hospital, Emergency Medicine Clinic, Istanbul, Turkey

## Abstract

There is no specific antidote for intoxication with synthetic cannabinoids. In this case series, we considered the efficiency of intravenous lipid emulsion therapy in four cases, who presented to emergency department with synthetic cannabinoid (bonzai) intoxication. The first patient had a GCS of 3 and a left bundle branch block on electrocardiography. The electrocardiography revealed sinus rhythm with normal QRS width after the treatment. The second patient had bradycardia, hypotension, and a GCS of 14. After intravenous lipid emulsion therapy, the bradycardia resolved, and the patient's GCS improved to 15. The third patient presented with a GCS of 8, and had hypotension and bradycardia. After the treatment, not only did the bradycardia resolve, but also the GCS improved to 15. The fourth patient, whose electrocardiography revealed accelerated junctional rhythm, had a GCS of 13. The patient's rhythm was sinus after the treatment. Cardiovascular recovery was seen in all four cases, and neurological recovery was also seen in three of them. Based on the fact that intravenous lipid emulsion is beneficial in patients intoxicated with lipophilic drugs, unstable patients presenting to the emergency department with acute synthetic cannabinoid intoxication may be candidates for intravenous lipid emulsion treatment.

## 1. Introduction 

Since their introduction in 2004, synthetic cannabinoid (SC) receptor agonists have become increasingly popular as an abused substance, especially among adolescents [1, 2]. Although they are most commonly named as “spice” collectively, “bonzai” is new and the most commonly preferred definition in Turkey [3, 4].

There is no specific antidote for SCs, and the treatment is mainly supportive. Popularity of intravenous lipid emulsion (ILE) therapy as a rescue antidote for the treatment of local anaesthetic toxicities has increased recently [5, 6]. ILE seems to be a new, safe, and promising treatment choice for SC intoxications.

In this case series, we aimed to discuss the efficiency of ILE therapy through the examination of four cases presented to the emergency department (ED) after bonzai (spice) consumption, which is known to be a lipophilic toxin.

## 2. Case 1

**Figure 1:**
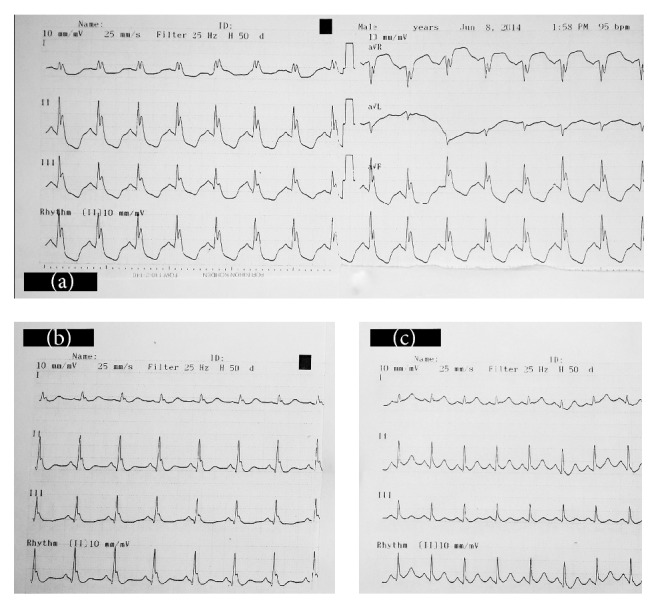
(a) Initial (before ILE treatment) electrocardiography of Case 1 with left bundle branch block. (b) Electrocardiography of Case 1 after bolus administration of intravenous lipid emulsion (5th minute of ILE). (c) Electrocardiography of Case 1 after intravenous lipid emulsion infusion (60th minute of ILE).

A thirty-five-year-old man who was found lying on the floor unconscious with empty “bonzai” bags near him was brought to the ED by his family. His medical history revealed that he had been a bonzai and heroin user for a while. The family refused the possibility of a suicide attempt and stated that the patient may have used heroin in addition to bonzai, but no other drugs. His Glasgow Coma Scale (GCS) was 3, pupils were miotic, and no anisocoria was observed. Arterial blood pressure (ABP) was measured as 110/75 mmHg, pulse rate was 95 beats/minute, body temperature was 36,9°C, and O_2_ saturation was 65%. His first electrocardiography (ECG) revealed a left bundle branch block (LBBB) (QRS complex width = 150 milliseconds) and sinus rhythm at a rate of 100 beats/minute (Figure 1). In the venous blood gases analyses (VBG) a respiratory acidosis was noted with a pH of 6.9 and paCO_2_ of 125 mmHg. Liver enzymes were elevated (aspartate transaminase = 806 U/L and alanine aminotransferase = 477 U/L) and so were the renal function tests (creatinine = 2,05 mg/dL, blood urine nitrogen = 53,5 mg/dL). White blood cell count was 25,6 K/uL and the international normalized ratio (INR) was 1,32. Other laboratory tests were normal.

As soon as the patient was intubated, a 1,5 mL/kg bolus of 20% lipid was administered intravenously, followed by an infusion of a 0,25 mL/kg/minute for 60 minutes (total dose of 1155 mL). Just after the bolus administration of ILE (5 minutes), narrowing of QRS complexes was observed. When the ILE infusion finished, the QRS complex was totally normal with width of 90 milliseconds (Figure 1). Despite the change in width of QRS, clinical improvement was not observed. Based on the suspicion of heroin use, naloxone was indicated, but we did not have the drug, nor did other hospitals in the city. After admission to the intensive care unit, the patient's renal function tests, liver enzymes, and coagulation parameters were all continued, increasing progressively. Acute respiratory distress syndrome (ARDS) also developed, and despite adequate fluid resuscitation, he became hypotensive and needed positive inotropic agents to maintain normal arterial pressure. He died two days later due to ARDS and multiorgan failure.

## 3. Case 2

**Figure 2:**
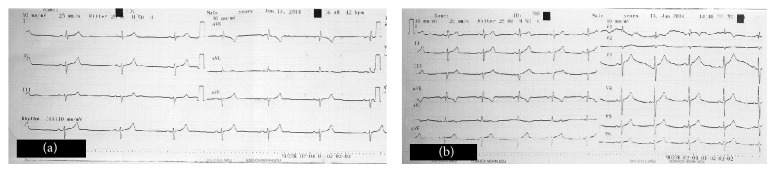
(a) Initial electrocardiography of Case 2 with sinus bradycardia. (b) Electrocardiography of Case 2, 45 minutes after initiation of intravenous lipid emulsion infusion.

A nineteen-year-old-man presented to the ED with a complaint of confusion after smoking bonzai. On examination, he had ABP of 70/30 mmHg, body temperature of 36,7°C, pulse rate of 42 beats/minute, GCS of 14, and he had no orientation and cooperation. His ECG revealed sinus bradycardia at a rate of 41 beats/minute (Figure 2). Other physical examination findings and laboratory tests were unremarkable. After infusion of 2000 mL of normal saline, hypotension persisted, and a 1,5 mL/kg bolus of 20% lipid, followed by an infusion of a 0,25 mL/kg/minute for 60 minutes, was administered (total dose of 990 mL). After bolus infusion of ILE (5 minutes) bradycardia started to resolve with a pulse rate of 50–55 beats/minute. When the infusion finished, the bradycardia had completely resolved, ABP was measured 110/70 mmHg, and the patient's GCS improved to 15 two hours after infusion. ILE was the only treatment performed for the symptomatic bradycardia. When he was completely conscious, his medical history was detailed and he confessed to the consumption of bonzai. He was discharged in good health after 24 hours of observation with no complications.

## 4. Case 3

**Figure 3:**
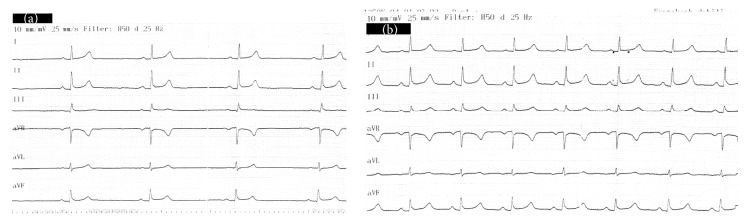
(a) Initial (before ILE treatment) electrocardiography of Case 3 with sinus bradycardia. (b) Electrocardiography of Case 3 after bolus infusion (5th minute of ILE) of intravenous lipid emulsion.

A fifteen-year-old unconscious man presented to the ED via ambulance. His medical history revealed that he became unresponsive after smoking bonzai. Both the patient and his friends denied use of other drugs or ingestion of any other substance. His ABP was 80/40 mmHg, pulse rate was 36 beats/minute, body temperature was 37,1°C, and O_2_ saturation was 94%. His initial GCS was 8 and pupils were mydriatic. His physical examination findings were totally normal except bradycardia, hypotension, and a low GCS score. His laboratory results were normal. The ECG showed a sinus bradycardia at a rate of 36/minute (Figure 3). We decided to give ILE when no response was observed after 2000 mL of normal saline solution. 1.25 mL/kg bolus of %20 lipid was administered and it was followed with infusion of 0.50 mL/kg/minute for 60 minutes (total dose of 2100 mL). No other drugs were given in addition to ILE. After ILE infusion, not only did the bradycardia resolve in five minutes, but also his GCS improved to 15 in two hours. The patient was observed for 24 hours in the ED and had no complications during this period. He was discharged in good health after the follow-up period.

## 5. Case 4

**Figure 4:**
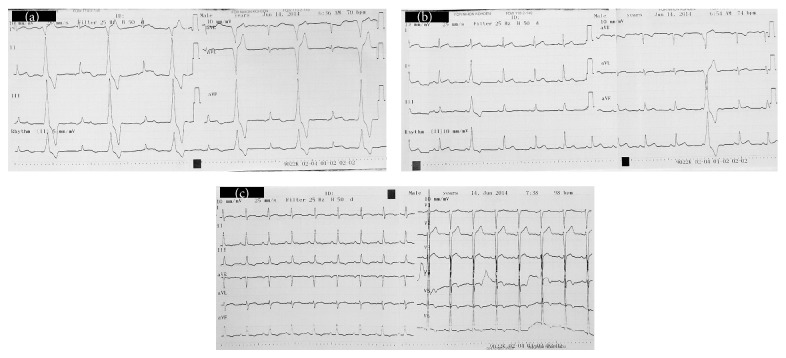
(a) Initial (before ILE treatment) electrocardiography of Case 4 with accelerated junctional rhythm concomitant with bigeminy ventricular extrasystoles. (b) Electrocardiography of Case 4 after bolus administration of intravenous lipid emulsion (5th minute of ILE). (c) Electrocardiography of Case 4 after intravenous lipid emulsion infusion (60th minute of ILE).

A seventeen-year-old man with confusion after cannabinoid consumption transferred to our ED from a rural hospital. His ABP was 115/68 mmHg, pulse rate was 80 beats/minute, O_2_ saturation was 98%, body temperature was 36,7°C, and he was disoriented with a GCS score of 13. Other physical examination findings were normal, and laboratory results were unremarkable. His ECG revealed accelerated junctional rhythm at a rate of 70 bpm concomitant with bigeminy ventricular extrasystoles (VES). We thought bonzai might be responsible from the abnormalities in ECG, and the patient was treated with 1.5 mL/kg bolus of 20% lipid intravenously, followed by an infusion of a 0.25 mL/kg/minute for 60 minutes (total dose of 990 mL). On the fifth minute of ILE infusion, it was observed that the frequency of VES decreased, and after finishing the ILE infusion (at 60th minute of treatment), the patient's rhythm was sinus at a rate of 74 beats/minute with no VES (Figure 4). His GCS also improved to 15 four hours after ILE infusion. He was discharged in good health after 24 hours of observation without any complications.

## 6. Discussion 

Synthetic cannabinoid receptor agonists are smokable herbal mixtures, marketed as legal marijuana in some countries [4]. They are commonly marketed as “legal highs.” SCs have increased in popularity, owing to their low costs and not being detected on traditional urine drug screens [7]. SC products have several street and commercial names such as spice, aroma, barely legal, Yucatan fire, dream, fusion galaxy, pep spice, gorilla, K2, and K3. Bonzai and Jamaican gold are the most popular names of SCs in Turkey [2–4]. In our four cases, all patients and their relatives named the SC used as bonzai.

Users report that “spice” has a stronger psychotropic effect than marijuana. The potency of being high on JWH-018 is 5 times that of THC, while AM-694 has 500 times more potency [3]. SCs are not detected in routine, traditional drug screens; this makes them popular in adolescents [8]. Yet, newer immunoassays with high sensitivity and specificity for rapid screening of SCs in human urine are developing due to the growing need for detecting new synthetic cannabinoids [9].

Synthetic cannabinoids can cause either decreased anxiety or dysphoric reactions, including anxiety and panic. Arterial hypertension and tachycardia are the most common cardiovascular side effects, but hypotension and bradycardia were also reported. The effects are seen after 0.5–2 hours of consumption, and the duration is prolonged [3, 10]. Two of our cases had bradycardia and hypotension, while ABPs and pulse rates of the other two were in normal range. GCSs of all four patients were under 15.

ILE has been reported to reverse cardiovascular collapse in overdoses of local anaesthetic agents, and it is endorsed as an antidote for systemic toxicity of local anaesthetics by many authors [11, 12]. Advanced Cardiac Life Support guidelines also recommend ILE for cardiac arrest, secondary not only to local anaesthetics, but also to beta blockers when conventional resuscitative therapies have failed. ILE is also thought to be an effective antidote for other lipophilic drug poisonings [13–15].

While ILE's exact mechanism is not known, the first and most widely accepted mechanism is “the lipid sink” theory. This was first presented by Weinberg et al. in 1998. Drugs that are free in the intravascular space are thought to be trapped within the ILE and this reduces the concentration and toxicity of the drug. Distribution of lipid soluble drugs from tissue to a circulating lipid phase also occurs [12, 16, 17]. In vitro studies support the lipid sink theory, while competing hypotheses and some in/ex vivo small animal studies suggest that a positive inotropic or metabolic effect underlies the dramatic effects of lipid therapy [18]. Some newer publications also have identified sink-independent effects and put forward alternative mechanisms such as hemodilution [19]. ILE not only does recover cardiovascular collapse but also reverses neurologic signs and symptoms of lipophilic drug intoxication [15].

We know that all four patients inhaled bonzai, and only in the first case did the patient have an uncertain history of heroin use in addition to bonzai. Both heroin and cannabinoids are extremely lipid soluble [10, 20]. Based on the fact that ILE is beneficial in intoxicated patients with lipophilic drugs we decided to treat these patients with ILE. In the previous literature, only Cevik et al. reported successful ILE treatment of a bonzai user with hypotension and a low GCS score [21]. Two of our patients with bradycardia, hypotension and low GCS scores, had normal pulse rates, ABPs, and GCSs soon after the ILE treatment. The ECG in Case 4 showed normal sinus rhythm after ILE treatment, while his initial ECG revealed junctional rhythm with bigeminy ventricular extrasystoles. His GCS was also improved from 8 to 15. Both cardiac and neurologic signs and symptoms of the 3 patients resolved after ILE therapy.

Only one of the four patients (Case 1) died despite ILE therapy. This patient had a different history of suspicious use of heroin in addition to bonzai. The major problem with the herbal compounds named as spice or bonzai is the uncertainty of what exactly they include. Gurdal et al. reported that 98.3% of 1200 herbal compounds named as bonzai contained synthetic cannabinoids. In their study, it is also stated that 1.7% of the herbal compounds included other psychoactive substances [4]. Furthermore, there may be many other unknown or little known chemicals in those herbal compounds. A recently published article on an internet news site about a synthetic cannabinoid cooker warns about the many risks users take with questionably made substances. According to the report, agents found rat poison among the ingredients the culprit used to make his own variety of the drugs [22]. We believe, in our first case, the patient might have died due to not only bonzai use, but also use of heroin or other chemicals whose ingredients he had little to no knowledge about. It is also known that ILE treatment has some complications like pancreatitis, acute lung injury, ARDS, and laboratory interference induced by lipemia [6]. ILE might have been responsible for ARDS developing in Case 1, but it is difficult to clinically discern whether ARDS is the result of the lipid or it is the result of critical illness. The patient was critically ill with a GCS score of 3 and deep respiratory acidosis. Even so, we saw the contribution of ILE treatment improved the ECG of the patient. His dramatically narrowed QRS complex demonstrated that administration of ILE might have helped the collapsed cardiovascular system to recover.

## 7. Conclusion

Based on the fact that ILE is beneficial to patients intoxicated with lipophilic drugs, unstable patients presented to the ED with acute SC intoxication may be candidates for ILE treatment.
